# Protocol to identify endogenous proximal proteins using biotinylation by antibody recognition

**DOI:** 10.1016/j.xpro.2024.103547

**Published:** 2025-01-10

**Authors:** Camilla Rega, Mercedes Pardo, Lesley-Ann Martin, Jyoti Choudhary

**Affiliations:** 1 The Institute of Cancer Research, 237 Fulham Road, London SW3 6JB, UK

**Keywords:** cell biology, protein biochemistry, proteomics

## Abstract

Biotinylation by antibody recognition (BAR) is an antibody-based approach for mapping proximal protein interactions in cells. Here, we present a protocol to biotinylate and identify proximal proteins using BAR. We describe steps for defining proximity labeling reaction conditions, assessing enrichment using western blot, and sample preparation for mass spectroscopy analysis. We then detail procedures for data analysis and identifying proximal proteins. This approach differs from standard proximity labeling techniques, which rely on genetically engineered enzymes fused to the target protein.

For complete details on the use and execution of this protocol, please refer to Rega et al.^1^

## Highlights

• Protocol for identifying proximal protein interactions using antibody recognition
• Outlines optimization steps via imaging and western blot before mass spectrometry
• Details proteomic sample preparation and data analysis processes

## Abstract (editor highlights)

Publisher’s note: Undertaking any experimental protocol requires adherence to local institutional guidelines for laboratory safety and ethics.

## Graphical abstract

**Figure:**
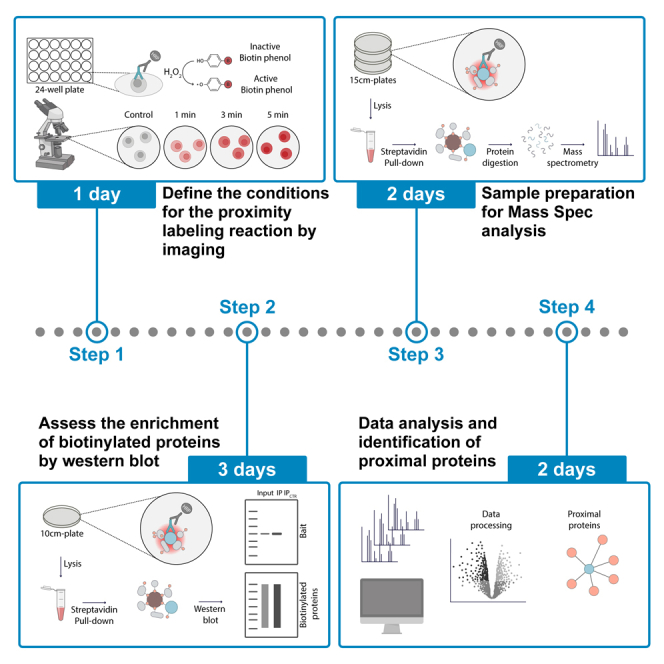

## Before you begin

Biotin proximity labeling is a widely used approach to detect proteins close to a protein of interest in living cells.^2^ This technology typically involves genetic manipulation to introduce a labeling enzyme fused to the target protein in cells. These enzymes convert inert biotin-substrates into reactive biotin-intermediates that covalently label proximal proteins. Biotin-tagged proteins are then enriched with streptavidin beads and identified by mass spectrometry. In the last decade proximity labeling has enabled the identification of numerous protein networks with remarkable spatial and temporal resolution. However, the genetic manipulation required to generate the target-enzyme fusion may not always be possible or desirable. In this context, Biotinylation by Antibody Recognition (BAR) has recently emerged to overcome this limitation.^3^^,^^4^ BAR replaces fusion enzymes with horseradish peroxidase (HRP)-conjugated antibodies specifically targeting a protein of interest through recognition. Upon the addition of hydrogen peroxide, the HRP enzyme converts biotin-phenol into highly reactive biotin radicals that covalently label tyrosine residues on proximal proteins, typically within a radius of 10 to <200 nm.

The protocol below was used in a recent publication by Rega and co-authors^1^ that investigated the Estrogen Receptor proximal proteome in MCF7 cells. This protocol has also been used to target various other endogenous proteins in different biological contexts and cell lines (unpublished data).

In designing the experiment for BAR mass spectrometry analysis, it is important to include a negative control sample that omits the primary antibody incubation. Where possible, use cells in which the expression of the target protein is silenced, and include both primary and HRP-conjugated antibodies. Alternatively, a reference control targeting a protein residing in a different cellular compartment can be used to filter out proteins that are not proximal to the target protein.

**Table 1:** Recommended buffer volumes based on experimental scale

–	Microscopy	Western blot	Mass spectrometry
Buffer	0.5 mL	5 mL	10 mL

## Key resources table

**Table:** 

REAGENT or RESOURCE	SOURCE	IDENTIFIER
**Antibodies**		

Anti-estrogen receptor monoclonal antibody (working dilution: 1:200)	Leica Biosystems	Cat# NCL-L-ER-6F11; RRID:AB_563706
Anti-mouse immunoglobulins/HRP antibody (working dilution: 1:2,000)	Dako	Cat# P0447; RRID:AB_2617137
Goat anti-mouse IgG (H + L) highly cross-adsorbed secondary antibody, Alexa Fluor Plus 488 (working dilution 1:500)	Thermo Fisher Scientific	Cat# A32723; RRID:AB_2633275

**Chemicals, peptides, and recombinant proteins**		

Acetonitrile, HPLC gradient grade	Fisher Scientific	Cat# A/0627/17
Biotin	Sigma-Aldrich	Cat# B4501
Biotin phenol	Iris Biotech	Cat# LS-3500
Bovine serum albumin (BSA)	Fisher Scientific	Cat# 11483823
DL-dithiothreitol	Sigma-Aldrich	Cat# D0632
DMSO	Sigma-Aldrich	Cat# D2650
Dried skimmed milk powder	N/A	N/A
Formaldehyde solution	Sigma-Aldrich	Cat# 252549
Formic acid, LC/MS grade	Fisher Scientific	Cat# 10596814
Hydrogen peroxide solution, 30% (w/w) in H_2_O	Sigma-Aldrich	Cat# H1009
Iodoacetamide (IAA)	Sigma-Aldrich	Cat# I1149
Phosphate-buffered saline (PBS)	N/A	N/A
Pierce Streptavidin magnetic beads	Thermo Fisher Scientific	Cat# 88817
Pierce Trypsin protease, MS grade	Thermo Fisher Scientific	Cat# 90057
Sodium chloride (NaCl)	N/A	N/A
Sodium L-ascorbate	Sigma-Aldrich	Cat# A7631
Streptavidin-horseradish peroxidase conjugate (working dilution: 1:1,000)	Thermo Fisher Scientific	Cat# S911
Streptavidin, Alexa Fluor 647 conjugate (working dilution: 1:1,000)	Thermo Fisher Scientific	Cat# S32357
TEAB Triethylammonium bicarbonate buffer, 1.0 M	Sigma-Aldrich	Cat# T7408
Tris (2-carboxyethyl) phosphine hydrochloride solution, 0.5 M, pH 7.0 (TCEP)	Sigma-Aldrich	Cat# 646547
Tris-buffered saline (TBS)	N/A	N/A
Triton X-100	Sigma-Aldrich	Cat# T9284
Tween 20	Sigma-Aldrich	Cat# P7949
Ultrapure SDS solution, 10%	Fisher Scientific	Cat# 24-730-020
Water, HPLC gradient grade	Fisher Scientific	Cat# W/0106/17

**Critical commercial assays**		

Pierce BCA Protein Assay Kits	Thermo Fisher Scientific	Cat# 23225

**Deposited data**		

The raw mass spectrometry data files generated for this project have been deposited to the ProteomeXchange Consortium via the PRIDE partner repository (https://www.ebi.ac.uk/pride/)	Rega et al.^1^	https://www.ebi.ac.uk/pride/archive/projects/PXD043294

**Experimental models: Cell lines**		

MCF7	ATCC	N/A

**Software and algorithms**		

Perseus	N/A	N/A
Proteome Discoverer	Thermo Fisher Scientific	N/A

**Other**		

24-well plates	Thermo Fisher Scientific	Cat# 150628
4x Laemmli sample buffer	Bio-Rad	Cat# 1610747
Cell lifter	Fisher Scientific	Cat# 9125
Centrifuge for 1.5 mL tubes	N/A	N/A
Glass coverslips	VWR	Cat# 31-0150P
Mounting medium with DAPI	3B Scientific	Cat# H-1200-10
Nupage 4 12% Bis Tris Protein Gels	Thermo Fisher Scientific	Cat# NP0321BOX
Pierce Spin cups and columns	Thermo Fisher Scientific	Cat# 69702
Pierce SuperSignal West Pico PLUS chemiluminescent substrate	Thermo Fisher Scientific	Cat# 34577
Protein LoBind micro tube 1.5 mL	Eppendorf	Cat# 0030108116
Sonicator with probe	N/A	N/A
Vacuum concentrator	N/A	N/A

## Materials and equipment

***Note:*** Buffer volumes should be determined based on the scale of the experiment. Refer to Table 1 for guidance on appropriate volumes relative to the number of samples and plate sizes. Adjust accordingly to ensure sufficient buffer preparation for your specific experimental setup.

***Note:*** For steps where the buffer volumes differ from those reported in the table, specific volumes are indicated within the protocol.

**Table:** PBST buffer

Reagent	Final concentration	Amount
Phosphate Buffered Saline (PBS)	N/A	500 mL
Tween-20 (100%)	0.05 (v/v)%	0.5 mL
ddH_2_O	N/A	–
Total	N/A	500 mL

Store at 18°C–22°C for up to six months.

**Table:** TBST buffer

Reagent	Final concentration	Amount
Tris Buffered Saline (TBS)	N/A	500 mL
Tween-20 (100%)	0.05 (v/v)%	0.5 mL
ddH_2_O	N/A	–
Total	N/A	500 mL

Store at 18°C–22°C for up to six months.

**Table:** Blocking buffer

Reagent	Final concentration	Amount
BSA	1%	5 g
PBST buffer	N/A	500 mL
Total	N/A	500 mL

Store at 4°C for up to one week.

**Table:** 200× biotin-phenol stock

Reagent	Final concentration	Amount
Biotin phenol	275 mM	250 mg
DMSO	N/A	2.5 mL
Total	N/A	2.5 mL

Store in 100 μL-aliquots at −80°C for up to one year.

**Table:** Biotinylation buffer

Reagent	Final concentration	Amount
200x biotin phenol	0.14 mM	5 μL
Hydrogen peroxide (30%)	0.03%	10 μL
PBST buffer	N/A	10 mL
Total	N/A	10 mL

Prepare fresh and add hydrogen peroxide before use.

**Table:** Quenching buffer

Reagent	Final concentration	Amount
Sodium ascorbate	0.5 M	50 g
PBST buffer	N/A	500 mL
Total	N/A	500 mL

Prepare fresh.

**Table:** Lysis buffer

Reagent	Final concentration	Amount
SDS (10%)	2%	2 mL
PBST	N/A	–
Total	N/A	10 mL

Store at 4°C for up to one week.

**Table:** Reducing buffer

Reagent	Final concentration	Amount
DTT (Dithiothreitol)	0.5 M	38.5 mg
TEAB	100 mM	0.5 mL
Total	N/A	0.5 mL

Store in aliquots at −20°C for up to one year.

**Table:** Alkylating buffer

Reagent	Final concentration	Amount
IAA (Iodoacetamide)	0.2 M	18 mg
TEAB	100 mM	0.5 mL
Total	N/A	0.5 mL

Prepare fresh or store at 4°C for short periods (typically no longer than a day). Keep in the dark.

## Step-by-step method details

### Define the conditions for the proximity labeling reaction by imaging

**Timing: 1 day**

This step enables the assessment of spatial specificity of the proximal biotinylation reaction in fixed cells using microscopy.

**CRITICAL:** Antibodies should be titrated for optimal resolution and minimal non-specific binding. Excess antibodies will bind promiscuously and result in background biotinylation. We recommend choosing monoclonal antibodies suitable for immunofluorescence or immunohistochemistry.

**CRITICAL:** Include a negative control sample omitting the primary antibody incubation. This should lead to absence of biotinylation. Where possible use cells in which the expression of the target protein is silenced and include both primary and HRP-conjugated antibody.

**CRITICAL:** Different labeling times should be tested to avoid excessive background biotinylation and to ensure that the biotin signal is comparable to the endogenous protein levels. Aim for a biotin signal that closely matches the intensity of the endogenous protein signal whenever possible.

***Note:*** The protocol described below is optimized for BAR in MCF7 cells grown on plates or coverslips and builds on previously published methods^3^ by providing optimized steps that critically improve the accuracy of BAR results. We also note that this protocol was efficiently performed on Retinal Pigment Epithelial (RPE1) cells.

1. Grow cells onto glass coverslips into 24-well plates to around 60% confluency in recommended cell culture medium.
2. Fix cells.
  ***Note:*** Percentage of formaldehyde and incubation time may vary based on cell type used. We recommend using the conditions typically used for immunofluorescence (IF) or immunohistochemistry (IHC) for the same cell type. Please note, Formaldehyde is toxic and corrosive. Avoid contact with skin and eyes. Use in a chemical fume hood.
  a. Remove culture medium by aspiration and discard.
  b. Wash cells twice with 500 μL of PBS.
  c. Discard buffer from last wash.
  d. Working in a fume hood, add 500 μL of 4% formaldehyde in PBS.
  e. Incubate for 10 min at 15°C–25°C.
  f. Wash cells twice with 500 μL of PBS, with no incubation required.
3. Permeabilize the cells.
  ***Note:*** Percentage of permeabilization agent and incubation time may vary based on cell type used. We recommend using the conditions typically used for immunofluorescence (IF) or immunohistochemistry (IHC) for the same cell type.
  a. Discard buffer from last wash.
  b. Add 500 μL of 0.5% Triton X-100 in PBST.
  c. Incubate for 7 min at 15°C–25°C.
  d. Wash cells twice with 500 μL of PBST, with no incubation required.
4. Inactivate endogenous peroxidases.
  ***Note:*** Dilute hydrogen peroxide just before use.
  a. Discard buffer from last wash.
  b. Add 500 μL of 0.5% hydrogen peroxide in PBST.
  c. Incubate for 10 min at 15°C–25°C.
  d. Wash cells twice with 500 μL of PBST, with no incubation required.
5. Use antibody recognition to direct HRP to the protein of interest.
  a. Discard buffer from last wash.
  b. Add 500 μL of blocking solution.
  c. Incubate at 15°C–25°C for at least 1 h.
    ***Note:*** Incubation time and temperature may need optimization. The antibody solution needs to cover your samples completely.
  d. Discard buffer from last step and incubate with primary antibody in 300 μL blocking solution at 15°C–25°C for 1 h.
  e. Remove primary antibody solution and gently wash the cells five times with 500 μL PBST, with no incubation required.
  f. Incubate cells with HRP-conjugated antibody (1:2000 dilution) in 300 μL blocking solution at 15°C–25°C for 1 h.
  g. Remove HRP-antibody solution and gently wash the cells five times with 500 μL PBST, with no incubation required.
    ***Note:*** Antibodies should be tested at the concentration recommended by the supplier and adjusted accordingly. Optimal incubation conditions may differ between antibodies.
6. Perform proximity labeling reaction.
  a. Prepare biotinylation buffer and quenching buffer.
  b. Discard buffer from last step.
  c. Incubate cells with 300 μL of biotinylation buffer for different time points to optimize the labeling time.
    ***Note:*** The optimal labeling reaction time must be determined by testing different labeling incubation time points (i.e., 0.5-1-3-5 min) and it is dependent on the expression levels of the target protein and the quality of the primary antibody (See troubleshooting for further guidance).
  d. Quickly remove the biotinylation buffer and immediately wash three times with 300 μL of quenching buffer, with no incubation required.
    **CRITICAL:** This step is time sensitive. Ensure timely removal of the biotinylation buffer to prevent over-labeling.
7. Prepare cells for immunofluorescence.
  a. Discard buffer from last step.
  b. Incubate with appropriate fluorophore-conjugated secondary antibodies (1:500) and streptavidin fluorophore-conjugate (1:1000) in 300 μL blocking solution at 15°C–25°C for 1 h.
  c. Discard buffer from last step and wash cells three times with 500 μL of PBST, with no incubation required.
  d. Mount coverslips onto a glass slide using mounting medium.

**Pause point:** Mounted coverslips can be stored in the dark at 4°C for at least a month.

8. Image the coverslips using a fluorescence microscope with appropriate filter settings.

**Figure 1:**
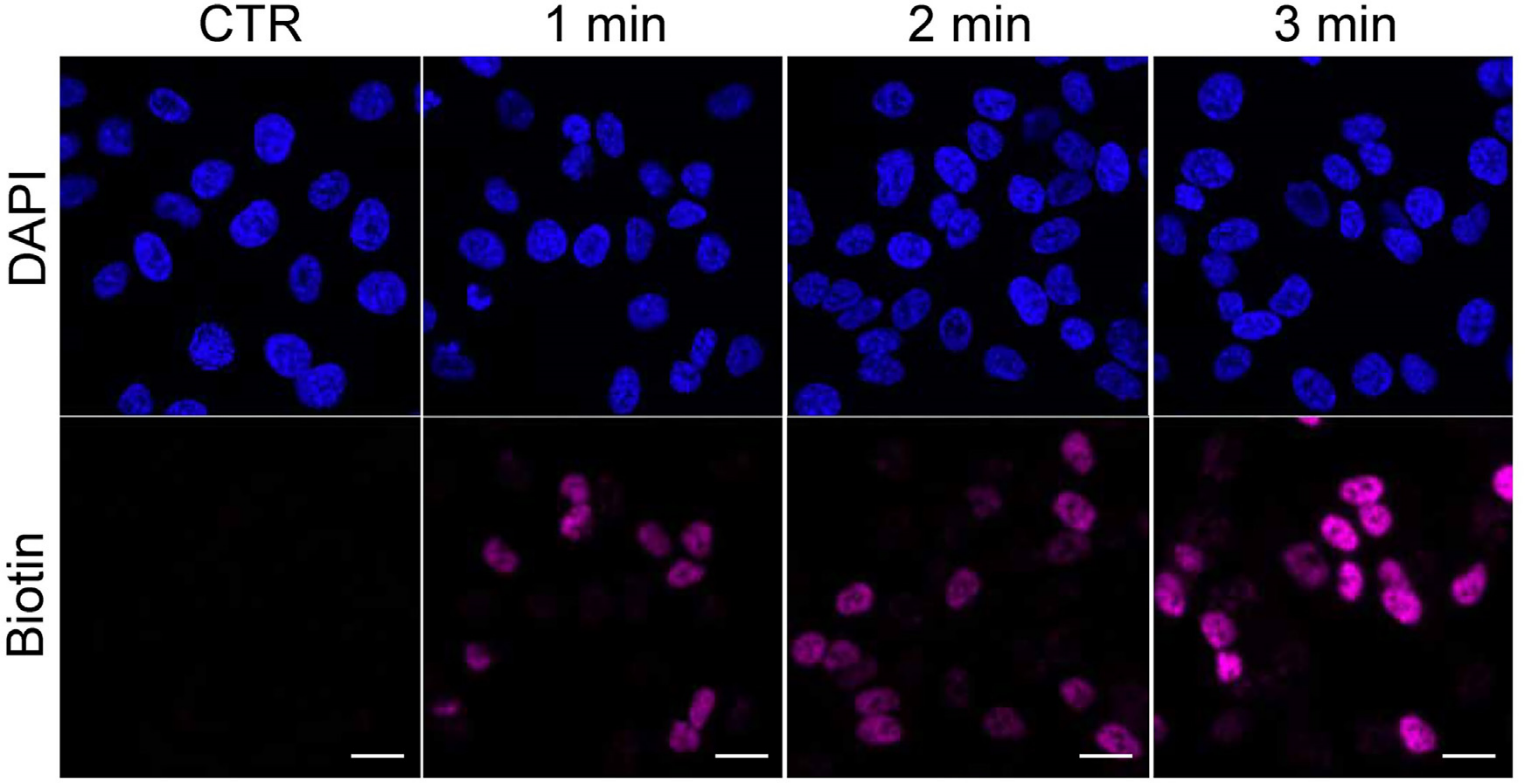
Optimizing the proximity labeling reaction time to target RB1 proximal proteins in RPE1 cells by imaging Confocal fluorescence imaging of RPE1 cells over a time-course to define the optimal labeling time. BAR was performed using an antibody targeting endogenous RB1 (sc73598 Santa Cruz) to biotinylate its proximal proteins at the indicated incubation time. Each sample was stained with DAPI to visualize the nuclei and Alexa Fluor Streptavidin 647 to visualize biotinylated proteins. Scale bar = 20 μm. The primary antibody was omitted in control samples. Note that proximity labeling reaction time is optimal at 3 min.

***Note:*** To achieve efficient and spatially restricted proximity biotinylation, it is essential to determine the optimal primary antibody concentration and labeling time that yield the most specific staining results with minimal background interference for each target protein (Figure 1). Troubleshooting 1 and 2.

### Assess the enrichment of biotinylated proteins by western blot

**Timing: 3 days**

In this step, a small-scale experiment is performed to assess the purification of biotinylated proteins. After performing the proximity labeling reaction, cells are lysed, biotinylated proteins enriched using streptavidin beads, eluted and analyzed by western blot. Carefully evaluate the extent and efficiency of the labeling reaction and optimize the ratio of streptavidin beads/lysate if required.

**CRITICAL:** Include a negative control sample omitting the primary antibody incubation. This should lead to absence of biotinylation. Where possible use cells in which the expression of the target protein is silenced and include both primary and HRP-conjugated antibody.

***Note:*** This protocol can also be performed on adherent cells detached by trypsinization. Compared to the procedure described in this protocol, cells are first detached with trypsin and then centrifuged for the subsequent steps to change buffers. This approach can be beneficial when high amounts of primary antibody are required, as it allows for smaller working volumes, thereby reducing the overall antibody usage.

9. Grow cells in 10-cm plates to around 80% confluency in recommended cell culture medium.

***Note:*** We use one 10-cm plate per condition; however, more plates may be required for different cell types or target proteins.

***Note:*** For steps detailed in the previous section, use a total volume of 5 mL per 10-cm plate.

10. Fix cells, as described in step 2.
11. Permeabilize the cells, as described in step 3.
12. Inactivate endogenous peroxidases, as described in step 4.
13. Use antibody recognition to direct HRP to the protein of interest.
  a. Discard buffer from last wash.
  b. Add 5 mL of blocking solution.
  c. Incubate at 15°C–25°C for at least 1 h.
  d. Discard buffer from last step and incubate with primary antibody in 5 mL blocking solution at 15°C–25°C for 1 h.
  e. Remove antibody solution and wash the cells three times with 5 mL PBST for 20 min each.
    ***Note:*** Prolonged washes are essential to ensure the removal of any residual antibody and avoid labeling background.
  f. Incubate cells with HRP-conjugated antibody (1:2000 dilution) in 5 mL blocking solution at 15°C–25°C for 1 h.
  g. Remove antibody solution and wash the cells five times with 5 mL PBST for 20 min each.
14. Perform proximity labeling reaction.
  a. Prepare biotinylation buffer and quenching buffer.
  b. Discard buffer from last step.
  c. Incubate cells with 1 mL of biotinylation buffer for the optimal time defined by imaging, gently swirling the plate throughout.
  d. Quickly remove the biotinylation buffer and immediately wash three times with 5 mL of quenching buffer, with no incubation required.

**CRITICAL:** Addition of quenching buffer to the cells is critical to stop the biotinylation reaction and prevent non-specific labeling. Work quickly and time the reaction accurately.

**Figure 2:**
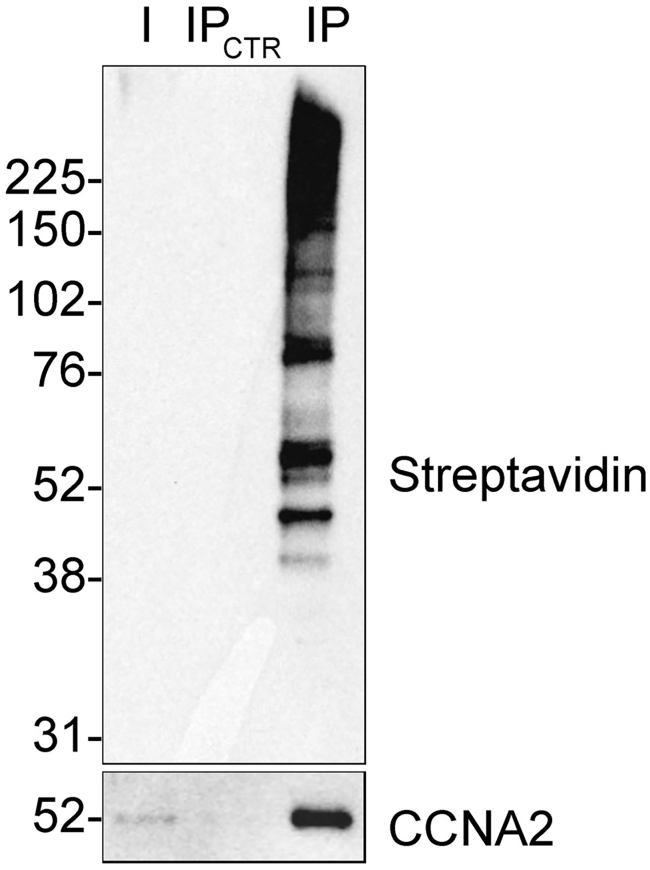
Assessing the biotinylated proteins enrichment by western blot BAR was performed using an antibody targeting endogenous CCNA2 (1:500, sc271682 Santa Cruz) to biotinylate its proximal proteins (3 min). The western blot shows that biotinylated proteins, including CCNA2, were efficiently enriched compared to the negative control. Western blot antibodies used: CCNA2 (1:500, sc271682, Santa Cruz) and Streptavidin HRP (1:1000, S911, Thermo Fisher Scientific).

15. Prepare cell lysate.
  ***Note:*** The sonication parameters may need to be adjusted until the cell lysate becomes clear. Keep the cells on ice during sonication.
  a. Discard buffer from last step.
  b. Wash cells three times with 5 mL of PBST, with no incubation required.
  c. Thoroughly discard buffer from last step.
  d. Add 100 μL of lysis buffer.
  e. Scrape cells and collect into microfuge tube.
    **Pause point:** Lysates can be stored at −80°C.
  f. Sonicate samples at 10% amplitude for 20 s, pause 20 s, and repeat once.
  g. Incubate lysates at 95°C for 10 min shaking.
    ***Note:*** Alternatively, when a sonicator is not available, cells can be lysed using a syringe with a 23-gauge needle or a homogenizer. After lysis, verify under a microscope that the cells have been efficiently lysed.
  h. Clear lysate by centrifugation 12000 *g* for 10 min at 15°C–25°C.
  i. Collect supernatant and discard pellet.
  j. Estimate the protein concentration using the BCA protein assay.
  k. Dilute the lysate to 1 mg/mL with PBST.
16. Enrich biotinylated proteins using streptavidin magnetic dynabeads.
  ***Note:*** The ratio of streptavidin dynabeads/lysate should be optimized to capture most of the biotinylated proteins while keeping the lowest volume of beads. We recommend starting with 25 μL streptavidin dynabeads/300 μg lysate.
  a. Wash the appropriate volume of streptavidin dynabeads three times.
    i. Transfer 25 μL of streptavidin dynabeads per condition to a 1.5 mL microfuge tube.
    ii. Place the tube on a magnetic rack for 30 s. Then, discard the supernatant and wash the beads three times with 500 μL PBST.
    iii. Discard buffer from last step.
  b. Add 300 μg lysate in 300 μL final volume with PBST (1 mg/mL final concentration).
  c. Place the tube on a magnetic rack for 30 s. Take 10 μL as input fraction.
  d. Incubate beads with lysate for 1 h at 15°C–25°C with rotation.
  e. Prepare washing buffers for step 16g.
  f. Place the tube on a magnetic rack for 30 s. Take 10 μL of supernatant as flow through fraction.
  g. Discard the rest of the supernatant and wash the beads:
    i. Once with 0.5% SDS in PBST.
    ii. Twice with 2 M NaCl in PBST.
    iii. Twice with PBST.
  h. Move beads to new tubes during the last wash step.
  i. Elute protein from beads by incubating each sample in 20 μL of 2x protein loading buffer supplemented with 2 mM biotin and 20 mM DTT at 95°C for 10 min with shaking.
  j. Pellet the beads using a magnetic rack and collect the eluate in new tubes.
  k. Add 10 μL of 4x protein loading buffer to the input and the flow-through fractions and incubate the samples at 95°C for 10 min.
  l. Run input, flow-through and eluate samples on a 4%–12% (v/v) Bis Tris protein gel.
  m. Transfer the proteins to a nitrocellulose membrane.
  n. Block with 5% (w/v) non-fat dry milk in 1x PBST while shaking at 15°C–25°C for 1 h.
  o. Wash the membrane three times with 1x PBST for 5 min each.
  p. To detect protein biotinylation, incubate membrane with streptavidin-HRP (1:1000) in 5% (w/v) BSA in 1x TBST at 15°C–25°C for 1 h or at 4°C for 12–18 h.
  q. Wash the membranes three times with 1x PBST for 5 min each.
  r. Develop the membranes using ECL western blotting substrate, then image.
    ***Note:*** Biotinylated proteins should be efficiently depleted from the flow-through fraction and enriched in the immunoprecipitated samples. Little or no biotinylation should be detected in control samples omitting the primary antibody (Figure 2).
    ***Note:*** We suggest probing the membrane for the target protein to assess if it was successfully biotinylated and enriched. However, this might not be detected if highly biotinylated. In this case, we recommend probing for well-known interactors instead.
    Troubleshooting 3 and 4 Western blot antibodies used: CCNA2 (1:500, sc271682, Santa Cruz) and Streptavidin HRP (1:1000, S911, Thermo Fisher Scientific).

### Sample preparation for mass spectrometry analysis

**Timing: 2 days**

This section outlines the protocol for preparing samples on a larger scale for mass spectrometry analysis, utilizing conditions optimized in the previous sections.

***Note:*** Three biological replicates are recommended for mass spectrometry analysis.

17. Grow cells in 15-cm plates around 80% confluency in recommended cell culture medium.

***Note:*** The number of cells required should be worked out after defining the amount of lysate required in the ‘assess the enrichment of biotinylated proteins by western blot’ section. In our experience, a minimum of 1 mg of lysate is sufficient for mass spectrometry experiments. However, this may vary depending on the expression levels of the target protein.

***Note:*** For steps detailed in the previous sections use a total volume of 10 mL per 15-cm plates unless otherwise specified.

18. Fix cells, as described in step 2.
19. Permeabilize the cells, as described in step 3.
20. Inactivate endogenous peroxidases, as described in step 4.
21. Use antibody recognition to direct HRP to the protein of interest, as described in step 13.
22. Perform proximity labeling reaction, as described in step 14.

***Note:*** Use 2 mL of biotinylation buffer per 15-cm plate.

23. Stop proximity labeling reaction.

**CRITICAL:** Addition of quenching buffer to the cells is critical to stop the biotinylation reaction and prevent non-specific labeling. Work quickly and time the reaction accurately.

24. Prepare cell lysate, as described in step 15.

***Note:*** Use 150 μL of lysis buffer per 15-cm plate.

**Pause point:** Lysates can be stored at −80°C.

25. Enrich biotinylated proteins using streptavidin magnetic dynabeads, as described in step 16.

***Note:*** We use 100 μL streptavidin dynabeads for 1 mg lysate. However, this ratio should be optimized as outlined in the ‘assess the enrichment of biotinylated proteins by western blot’ step of the before you begin section.

***Optional:*** Aliquots can be taken for western blot validation to confirm the efficiency of the protocol before mass spectrometry analysis.

26. Prepare samples for mass spectrometry.
  a. Resuspend beads in 100 μL of 100 mM TEAB.
  b. Add 4 μL of reducing buffer and incubate at 15°C–25°C for 10 min at 1000 g.
  c. Add 6 μL of alkylating buffer and incubate at 15°C–25°C in the dark for 45 min at 1000 g.
  d. Add 10 μL 0.2 mg/mL trypsin (2 μg) and incubate at 37°C for 12–18 h at 1000 g.
  e. Add an additional 2 μg of trypsin to the sample and incubate at 37°C for 3 h at 1000 g.
  f. Pellet beads by placing the tubes on the magnetic rack and transfer supernatant to a new tube.
  g. After digestion, acidify the sample by adding formic acid to 1% (v/v) final concentration.

***Optional:*** Isobaric mass tags (such as tandem mass tagging (TMT) can be used after this step to enable quantitative comparison.

27. Perform sample cleanup to remove contaminants and impurities before mass spectrometry analysis.

***Note:*** Various methods can be employed for sample cleanup, we have successfully used Cellulose Acetate Filter Spin Cups or C18 tips for this purpose, following the manufacturer’s instructions.

28. Dry out samples in a vacuum concentrator.
29. Run samples by LC-MS/MS.

***Note:*** We generally run our samples in an Orbitrap Fusion Lumos hybrid mass spectrometer coupled with an Ultimate 3000 RSLCnano UPLC system, operated in data-dependent acquisition (DDA) mode, using an 88 min method with a linear gradient of 4%–32% ACN/0.1% FA in 90 min at a 300 nL/min flow rate, total cycle time of 120 min for each fraction.

***Note:*** When labeling samples with TMT, we recommend fractionating the pooled samples using high-pH reversed-phase chromatography before mass spectrometry for optimal results.

### Data analysis and identification of proximal proteins

**Timing: 2 days**

In this section we describe the pipeline to analyze the MS raw data, identify putative proximal proteins and efficiently distinguish these from background binders.

30. Search the mass spectrometry data with the Proteome Discoverer 2.4 software using Sequest HT search engine against the reviewed UniProt database of *Homo sapiens*. The parameters are listed in Table 2.

***Note:*** Other software options, such as MaxQuant (freely available), can also be used for mass spectrometry data analysis.

31. Analyze the mass spectrometry data.
  ***Note:*** We typically use the Perseus software for downstream statistical analysis and visualization. Other freely available software tools for this analysis include LFQ-Analyst (https://doi.org/10.1021/acs.jproteome.9b00496) and FragPipe-Analyst (https://doi.org/10.1021/acs.jproteome.4c00294).
  a. Transform Label-Free Quantification (LFQ) values to log_2_.
  b. Normalize values by the median.
  c. Impute missing values.
    ***Note:*** In Perseus software the missing values are replaced by random numbers drawn from a normal distribution of 1.8 standard deviation down shift and with a width of 0.3 of each sample.
  d. Perform student’s *t* test comparing BAR-derived biotinylation with negative control samples.
  e. Determine cut-off value for high-confidence proximal proteins.
    ***Optional:*** A Receiver Operating Characteristic (ROC)-based analysis can be employed to define cut-off values, as previously described.^5^

**Table 2:** Parameters for searching the data with Proteome Discoverer

Parameter	Value
Variable modifications	Deamidated (N, Q), Oxidation (M) and acetylation (Protein N-terminus) Biotinylation (Y;(C18H23N3O3S))
Fixed modification	Carbamidomethyl (C)
Maximum missed cleavages	2
Precursor mass tolerance	20 ppm
Fragment mass tolerance	0.5 Da
Peptide False Discovery Rate (FDR)	0.01

## Expected outcomes

**Figure 3:**
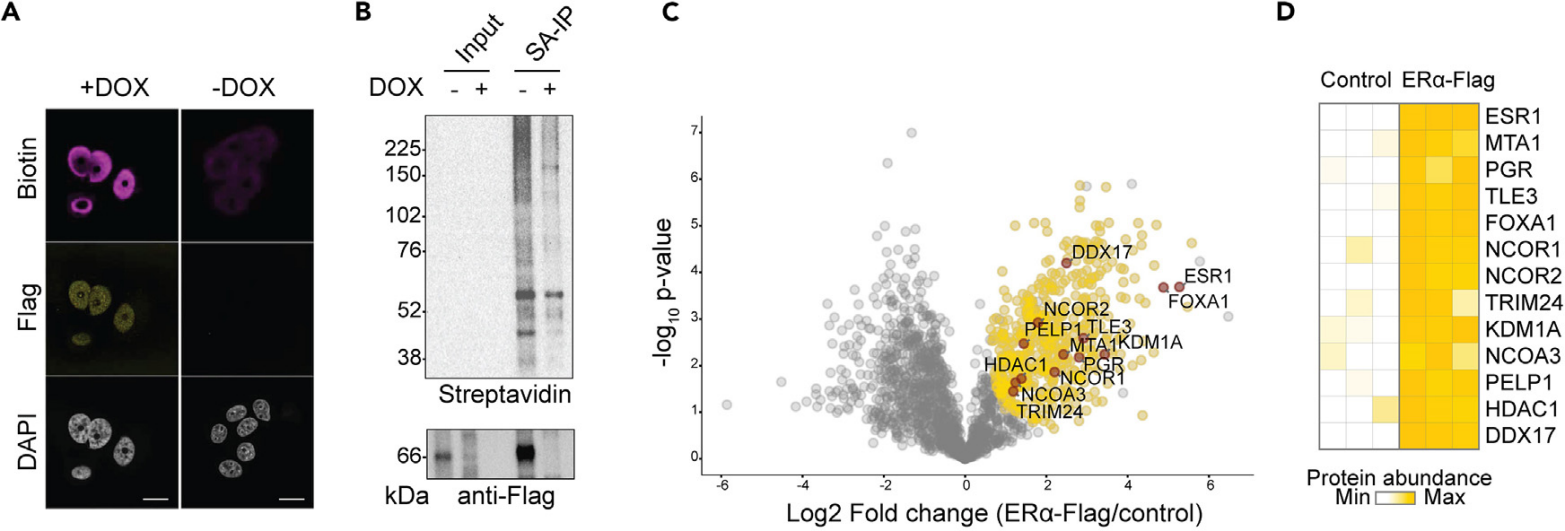
Representative result of expected outcome Cells stably expressing ERα-Flag under the control of a Tet-Off promoter (MCF7-TO cells) were labeled with biotin using Flag tag antibody (1:500, F1804 Sigma-Aldrich) to drive the proximity reaction. Cells were treated with doxycycline (DOX) to silence ERα-Flag expression in control samples. (A) Immunofluorescence images of ERα-Flag BAR. Biotin colocalized with ERα-Flag and biotinylation resulted in nuclear localization. Little biotinylation was observed in the negative control. Nuclei were detected by DAPI (gray); ERα-Flag was visualized by Alexa Fluor 488 dye (gold). Biotinylated proteins were detected by streptavidin-conjugated Alexa Fluor 647 dye (magenta). Scale bar: 20 μm. (B) Western blot analysis of ERα-Flag BAR biotinylated proteins. Labeled cells were lysed and incubated with streptavidin beads. ERα-Flag was efficiently immunoprecipitated (SA-IP) among biotinylated proteins (Input). Western blot antibodies used: anti-Flag (1:1000, #F1804, Sigma-Aldrich) and Streptavidin HRP (1:1000, S911, Thermo Fisher Scientific). (C) Volcano plot showing proteins significantly enriched in biotinylated versus control samples. Statistically significant proteins (student t test q-value <0.01) are shown in gold; proteins non-statistically significant are shown in gray. ERα well-known interacting proteins are highlighted. (D) Heatmap showing ERα known interactors enriched versus negative control. Proteins are colored according to their abundance.

The protocol is designed to biotinylate proteins proximal to a protein of interest using HRP conjugated antibody, bait recognition is used to anchor the HRP enzymatic catalysis of biotin phenol to generate short lived reactive species that labels tyrosine residues in its vicinity. Biotinylated proteins are then purified by affinity capture and identified by mass spectrometry. A successful experiment should lead to biotinylation levels comparable to the expression and localization of the target protein (Figure 3A). Known binding partners and proteins residing in the same subcellular localization or protein complex should be detected among the enriched biotinylated proteins (Figures 3B, 3C, and 3D). Negative control samples omitting primary antibody should lead to very low biotinylation levels and enable the identification of proteins non-specifically binding to the beads, as well as non-specific biotinylation caused by the HRP-conjugate antibody promiscuous binding. Tyrosine modification with biotin is not always detected and can vary depending on experimental conditions, including target protein expression levels, number of exposed tyrosine residues and antibody efficiency.

## Limitations

Since the outcome of this protocol depends on the efficiency of the antibody recognition, a critical step is identifying a suitable antibody targeting the protein of interest. The biotinylation extent of proximal proteins is dependent on the expression level of the target protein. Since low expressed proteins result in low biotinylation, a larger amount of starting material may be necessary. Maintaining levels of biotinylation similar to endogenous protein levels is essential to avoid non-specific labeling.

Furthermore, it is important to note that HRP enzymes catalyze the oxidation of short-lived biotin-phenol radicals that react with electron-rich amino acids, primarily tyrosine, on neighboring proteins resulting in their covalent tagging with biotin. Therefore, protein biotinylation efficiency will be affected by the number of accessible tyrosine residues.

Proximity labeling methods are known to result in a higher number of hits compared to other interaction proteomics approaches. This is because they detect also weak interacting partners and neighboring proteins, in addition to relatively stable physical binders. Therefore, appropriate negative controls should be included in the experimental design to efficiently remove non-proximal proteins. These include omitting the primary antibody, using cells that do not express the protein of interest (knock out cells, protein silencing) and targeting a protein residing in a different cellular compartment.

## Troubleshooting

### Problem 1

Biotinylation is not spatially restricted to the localization of the target protein.

### Potential solution

We strongly recommend using monoclonal antibodies suitable for immunofluorescence or immunohistochemistry. Moreover, antibodies should be titrated for optimal resolution and minimal non-specific binding. Excess antibodies will bind promiscuously and result in background biotinylation. Test different antibodies for the protein of interest by imaging and select the one that leads to the most specific staining. If possible, ideally use a cell line lacking the protein of interest to confirm the specificity of the primary antibody. We also found that different HRP-conjugated antibodies result in different biotinylation efficiency and patterns. We recommend using the HRP-conjugated antibodies indicated in the key resources table for optimal results.

### Problem 2

Biotinylation levels are higher than endogenous protein expression.

### Potential solution

This problem is mostly likely due to the proximity labeling reaction time. We found that 3 min is generally enough to achieve good biotinylation levels. However, high-abundant proteins may require lower incubation time, starting with 30 s. Alternatively, a lower concentration of biotin-phenol can be used in the biotinylation reagent. Finally, make sure that all reagents and solutions used are freshly made; particular attention should be given to the biotinylation buffer and quenching buffer.

### Problem 3

Poor enrichment of biotinylated proteins.

### Potential solution

If the levels of biotinylation are low, this can be due to insufficient amount of sample used for the enrichment. Try to increase the amount of starting material. If the levels of biotinylation are high, and biotinylated proteins are lost in the flowthrough, increase the amount of streptavidin beads to efficiently capture biotinylated proteins.

### Problem 4

Target protein not detected among the biotinylated proteins.

### Potential solution

We recommend using 25 μL streptavidin dynabeads for 300 μg lysate. However, the amount of lysate used for the enrichment of biotinylated proteins can be increased. If there is good enrichment of biotinylated proteins but the target protein is not detected by western blot, this can be because the target protein is highly biotinylated and thus may not be recognized by the antibody used for western blot. In this case, we suggest probing for known interacting partners instead. Alternatively, if the enrichment of biotinylated proteins is not efficient, the protocol should be optimized further (see problem 3).

### Problem 5

High background signal in negative controls.

### Potential solution

This is likely due to high non-specific binding of non-proximal proteins to the beads. We suggest trying more stringent washing conditions (including detergents) or increasing their number and duration.

## Resource availability

### Lead contact

Further information should be directed to and will be fulfilled by the lead contact, Camilla Rega (Camilla.rega@icr.ac.uk).

### Technical contact

Questions about the technical specifics of performing the protocol should be directed to and will be answered by the technical contact, Camilla Rega (Camilla.rega@icr.ac.uk).

### Materials availability

This protocol did not generate new unique reagents.

### Data and code availability

The raw mass spectrometry data generated for this protocol have been deposited to the ProteomeXchange Consortium via the PRIDE partner repository with the dataset identifier PXD043294.

## Acknowledgments

We thank Daniel Z Bar for expert assistance related to the BAR approach. We thank the Proteomics and Light Microscopy and Confocal Microscopy facilities at the ICR. This work was funded by the Arthur Foundation and Breast Cancer Now.

## Author contributions

Conceptualization, C.R. and J.C.; methodology, C.R.; investigation, C.R.; writing, C.R. and M.P.; visualization, C.R.; writing – review and editing, C.R., M.P., J.C., and L.-A.M.; funding acquisition, L.-A.M.; resources, L.-A.M. and J.C.; supervision, C.R., J.C., and L.-A.M.

## Declaration of interests

The authors declare no competing interests.
